# Altered Patterns of Fungal Keratitis at a London Ophthalmic Referral Hospital: An Eight-Year Retrospective Observational Study

**DOI:** 10.1016/j.ajo.2016.05.021

**Published:** 2016-08

**Authors:** Hon Shing Ong, Simon S.M. Fung, David Macleod, John K.G. Dart, Stephen J. Tuft, Matthew J. Burton

**Affiliations:** a Moorfields Eye Hospital NHS Foundation Trust, London, United Kingdom; b National Institute of Health Research (NIHR) Biomedical Research Centre at Moorfields Eye Hospital NHS Foundation Trust and the UCL Institute of Ophthalmology, London, United Kingdom; c Department of Infectious Disease Epidemiology, London School of Hygiene and Tropical Medicine, London, United Kingdom; d International Centre for Eye Health, London School of Hygiene and Tropical Medicine, London, United Kingdom

## Abstract

**Purpose:**

In previous studies of fungal keratitis (FK) from temperate countries, yeasts were the predominant isolates, with ocular surface disease (OSD) being the leading risk factor. Since the 2005–2006 outbreak of contact lens (CL)-associated *Fusarium* keratitis, there may have been a rise in CL-associated filamentary FK in the United Kingdom. This retrospective case series investigated the patterns of FK from 2007 to 2014. We compared these to 1994–2006 data from the same hospital.

**Design:**

Retrospective observational study.

**Methods:**

All cases of FK presenting to Moorfields Eye Hospital between 2007 and 2014 were identified. The definition of FK was either a fungal organism isolated by culture or fungal structures identified by light microscopy (LM) of scrape material, histopathology, or in vivo corneal confocal microscopy (IVCM). Main outcome measure was cases of FK per year.

**Results:**

A total of 112 patients had confirmed FK. Median age was 47.2 years. Between 2007 and 2014, there was an increase in annual numbers of FK (Poisson regression, *P* = .0001). FK was confirmed using various modalities: 79 (70.5%) by positive culture, 16 (14.3%) by LM, and 61 (54.5%) by IVCM. Seventy-eight patients (69.6%) were diagnosed with filamentary fungus alone, 28 (25%) with yeast alone, and 6 (5.4%) with mixed filamentary and yeast infections. This represents an increase in the proportion of filamentary fungal infections from the pre-2007 data. Filamentary fungal and yeast infections were associated with CL use and OSD, respectively.

**Conclusions:**

The number of FK cases has increased. This increase is due to CL-associated filamentary FK. Clinicians should be aware of these changes, which warrant epidemiologic investigations to identify modifiable risk factors.

Fungal keratitis (FK) is an important cause of infectious keratitis and ocular morbidity worldwide. The clinical diagnosis of FK can be difficult or may not initially be considered, resulting in delayed treatment. The available drugs have variable impact and in nonresponsive cases, surgical excision of the infected cornea is required to control the infection.^1^, ^2^, ^3^, ^4^ Moreover, visual outcomes of FK are often worse than for bacterial keratitis.^5^, ^6^

In tropical countries, fungi account for up to 67% of corneal infections.^7^, ^8^, ^9^, ^10^, ^11^, ^12^, ^13^, ^14^ Filamentary fungi associated with trauma cause the majority of these infections. This has also been reported in regions with subtropical climates.^5^, ^15^, ^16^ In contrast, FK is a relatively rare clinical problem in temperate countries such as the United Kingdom (UK). A 2-year (2003–2005) national surveillance study in the UK estimated a minimum average incidence of FK at 0.32 (95% confidence interval [CI] 0.24–0.44) cases per million individuals per year.^17^ In that study, 23 of 39 (57.5%) were yeast (candida) infections and 17 of 39 (42.5%) were filamentary fungal infections. The majority of FK cases (25/39, 63.2%) were associated with ocular surface disease (OSD). This association was particularly strong for yeast infections, which all had OSD. Trauma was associated with 11 of 39 (29%) and contact lens (CL) use was reported in only 3 of 39 cases (7.9%), which were all filamentary. This was consistent with reports of FK in other high-income countries with temperate climates.^18^, ^19^, ^20^, ^21^, ^22^, ^23^, ^24^, ^25^, ^26^ An earlier study from Moorfields Eye Hospital, a large ophthalmic hospital serving London and southeast England, reported on FK presenting over a 13-year period (1994–2006).^27^ During that time, yeast infections accounted for the majority of FK cases and were strongly associated with OSD. During the same period CL-associated filamentary fungal keratitis was relatively uncommon.

In 2005–2006, there was an outbreak of *Fusarium* keratitis in CL users, reported in the United States, Singapore, Hong Kong, Europe, and the French West Indies.^28^, ^29^, ^30^, ^31^, ^32^, ^33^, ^34^ Cases were thought to be associated with the use of the ReNu with MoistureLoc (Bausch & Lomb, alexidine dihydrochloride 0.00045%) CL disinfection solution. Since this solution has been globally recalled on May 15, 2006, the incidence may have reduced.^29^ However, the perception among clinicians in the UK is that the number of FK cases has increased again in recent years, with proportionately more CL-associated filamentary fungi. To investigate this we conducted a retrospective study of all confirmed cases of FK presenting to Moorfields Eye Hospital from 2007 to 2014. We also compared these data with data collected from the same institution between 1994 and 2006.

## Methods

This study was a retrospective review of medical records. The protocol was approved by the Moorfields Eye Hospital Clinical Research Management and Audit Department (reference: CA13/CED/12). The study adhered to the tenets of the Declaration of Helsinki.

### Case Ascertainment

To identify cases we made the assumption that all cases of fungal keratitis (suspected or confirmed) would be commenced on topical antifungal treatment. We queried the hospital pharmacy database for the record numbers of all patients who were dispensed any antifungal treatment between January 1, 2007 and December 31, 2014. The medical notes were retrieved and reviewed to ascertain whether the individual met the case definition for inclusion in this study.

### Case Definition

For the purpose of this study we considered an individual with clinical features of suppurative keratitis to be a case of FK if the individual met 1 or more of the following criteria: (1) a fungal organism grown from a corneal scrape or biopsy sample on 1 or more culture media; (2) fungal elements present in light microscopy of a corneal scrape sample; (3) fungal elements present in histopathology of corneal biopsy tissue; (4) fungal elements identified by in vivo confocal microscopy (IVCM).

### Data Collection

Data collected from the medical record included: demographic information, country where FK was acquired, potential risk factors (such as CL use, topical steroid use, trauma), past ophthalmic history, microbiology results, IVCM results, and visual acuity outcome. The presenting visual acuity (VA) was routinely measured with a Snellen chart at 6 meters, with spectacle correction if available. The OSD group included patients with dry eyes, corneal exposure, blepharitis, persistent epithelial defects, or chronic ocular surface inflammatory conditions (eg, atopic keratoconjunctivitis, mucous membrane pemphigoid, Stevens-Johnson syndrome).

### Laboratory Investigations

At Moorfields, specimens for culture are typically obtained by scraping the base and edges of corneal ulcers with a sterile, disposable 23 gauge needle. Samples are smeared on a glass slide and stained for microscopy. Samples are also inoculated onto a minimum of blood agar and Sabouraud dextrose agar. In addition, several other media are also frequently used: Robertson's cooked meat broth, brain-heart infusion, and non-nutrient agar. All solid media are incubated at 37 C for a minimum of 1 week. Broths are usually cultured for 2 days and are then subcultured on solid media for a minimum of 1 week. All microbiological investigations were undertaken independently in an external laboratory and cultured isolates are sent to the UK Mycology Reference Laboratory for speciation. For biopsies, a superficial lamellar disc of affected cornea was taken under local anesthesia and sent for microbiological and histopathologic staining.^35^ Specimens from biopsy or keratoplasty material were cultured from homogenized tissue. The use of a panfungal polymerase chain reaction (PCR) assay only entered our routine practice during the last 24 months of the period studied. Therefore, for consistency throughout the 2007–2014 study period it was not used as an inclusion criterion for FK.

### In Vivo Confocal Microscopy

IVCM was performed and interpreted by experienced clinicians using the HRT II/RCM confocal microscope (Heidelberg Engineering, Dossenheim, Germany).^36^ Typically, the ulcer is systematically surveyed around its circumference and more centrally at variable depths to assess different levels of the cornea. Volume scans of areas of interest were captured and stored for analysis.

### Statistical Analysis

Data were managed in Access (Microsoft) and analyzed using STATA 13 (StataCorp LP, Texas, USA). Differences in the distribution of categorical variables between groups were analyzed using the χ^2^ test or logistic regression. For continuous variables, differences in distribution between groups were analyzed by the Mann-Whitney test. Poisson regression was used to analyze change in presentation rates. Multivariable logistic regression was used to investigate risk factors for filamentary vs yeast infection. Variables with significant associations on univariable analyses at a level of *P* < .05 were included in the initial multivariable analysis. Nonsignificant terms were then removed from the multivariable model in a step-wise manner, with only those with *P* < .05 being retained.

To assess longer-term trends we compared the 2007–2014 data with data collected and previously reported from the same hospital during the preceding 13 years (1994–2006).^27^ This is a subset of the previously reported data that only includes whole calendar years. The 1994–2006 series had more restricted inclusion criteria, as it only included cases that were both UK-acquired and culture positive. It pre-dated the routine use of IVCM. To compare data from the 2007–2014 series with the 1994–2006 series, we excluded cases in the 2007–2014 series that were acquired outside the UK or did not have a positive culture.

## Results

### Patient Characteristics

Between January 1, 2007 and December 31, 2014, 200 patients were prescribed an antifungal treatment for suspected FK. Of these, 112 patients met the study inclusion criteria for FK, representing an average of 14 cases per year. Of the 88 patients who were started on antifungal treatment but did not meet the inclusion criteria for confirmed FK, 30 received continued empirical treatment for fungal keratitis, 24 received continued treatment for a microbial keratitis of unspecified cause, 29 were diagnosed with bacterial keratitis, 3 were diagnosed with herpetic keratitis, 1 was diagnosed with mycobacterium keratitis, and 1 was diagnosed with a rheumatoid corneal melt.

**Figure 1:**
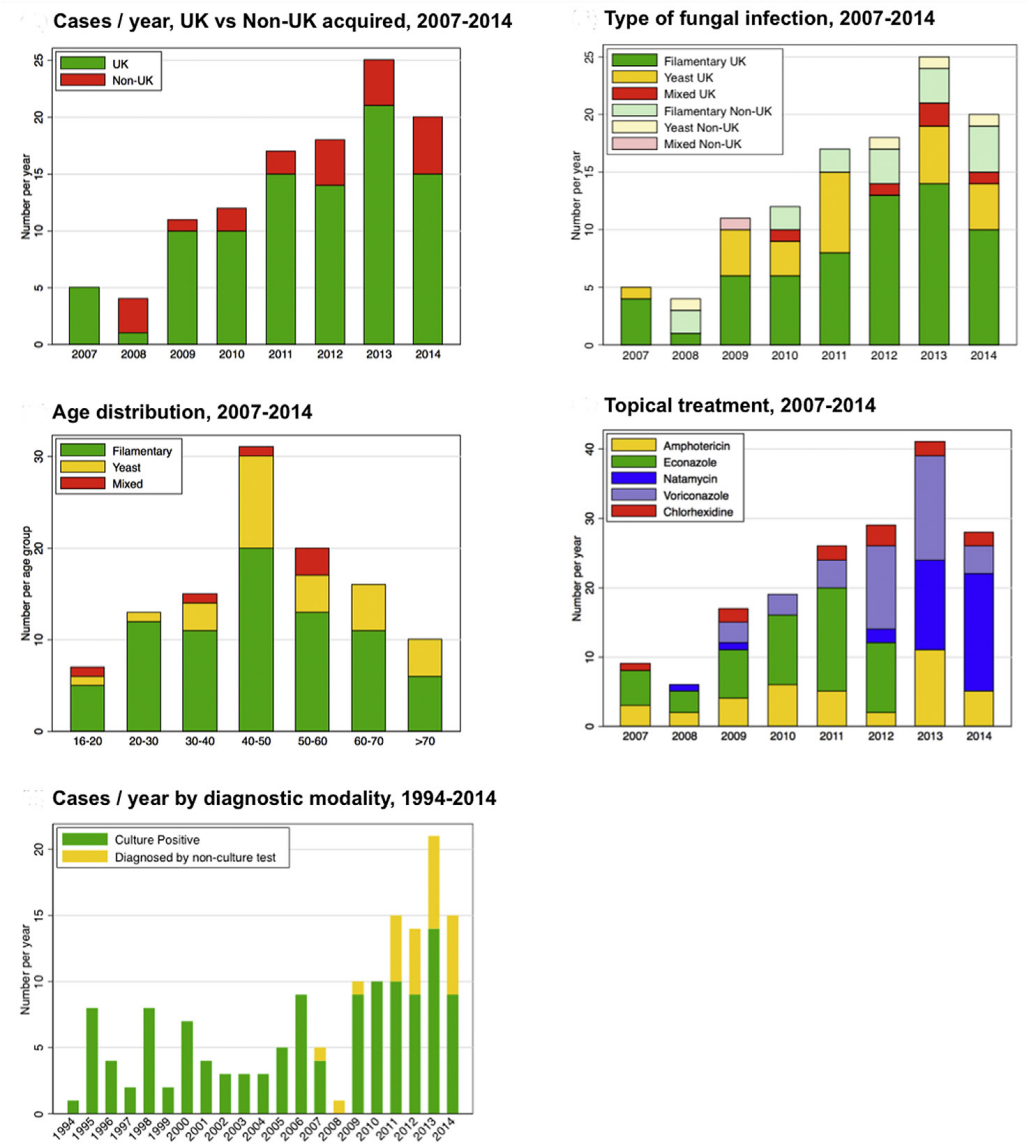
Patterns of fungal keratitis observed in this study. (Upper left) Number of cases of fungal keratitis presenting each year (2007–2014) subdivided into those acquired in the United Kingdom (UK) (91) and outside the UK (21). There was an increase with time in cases acquired in the UK (Poisson regression *P* = .0001) but not from outside the UK (Poisson regression *P* = .6). (Upper right) Number of cases of filamentary fungal infections, yeast infections, and mixed infections (filamentary and yeast) by year of presentation. There was an increase with time in the number of cases of filamentary fungi acquired in the UK: Poisson regression *P* = .005. There were no other statistically significant changes with time. (Middle left) Number of cases of filamentary fungal infections, yeast infections, and mixed infections (filamentary and yeast) by age group. (Middle right) Number of cases treated by different topical antifungal drugs, by year of presentation. (Lower left) Number of cases acquired in the UK only, 1994–2014, subdivided by a culture/nonculture diagnosis. There was an increase in the number of culture-positive, UK-acquired cases between 1994 and 2014: Poisson regression *P* = .003.

All patients had unilateral infections. Their median age was 47.2 years, and 46/112 (41.1%) were male (Table 1). The majority of cases were acquired in the UK (91/112 [81.3%]), largely from London and the east and the southeast of England (Table 2). Of the 112 cases, 21 were acquired outside the UK: 5/112 (4.5%) in other European countries, 3/112 (2.7%) in African countries, 7/112 (6.2%) in Asia, and 6/112 (5.4%) in the Americas (Table 2). Overall, there was strong evidence of an increase in the total annual number of cases presenting during the 2007–2014 period (Poisson regression, *P* = .0001, Figure 1, Upper left). When individuals who acquired their infections outside the UK are excluded from this analysis, there was still strong evidence of an increase in the annual number of cases (Poisson regression, *P* = .0001, Figure 1, Upper left). There was no evidence of an increase in the number of infections acquired outside the UK during the 2007–2014 period (Poisson regression, *P* = .6, Figure 1, Upper left).

### Diagnostic Modality

**Figure 2:**
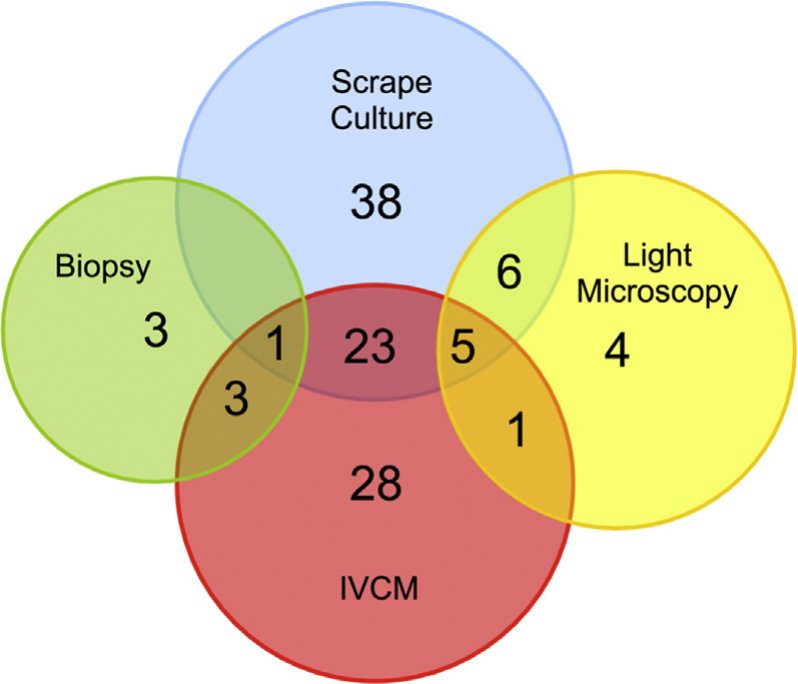
Diagnostic modality by which a diagnosis of fungal keratitis was made for the 112 cases. One biopsy was from a full excision biopsy at the time of penetrating keratoplasty. IVCM = in vivo confocal microscopy.

Figure 2 illustrates the diagnostic modality by which the FK diagnosis was made for the 112 cases. There was a positive culture in 79 patients: 73/112 (65.2%) from corneal scrapings, 6/112 (5.4%) from corneal biopsy culture. One was culture positive from both corneal scraping and biopsy samples. There was 1 case in which the presence of fungal hyphae was only confirmed by histopathology of the corneal button (biopsy) removed following a penetrating keratoplasty. In 16 of the 112 patients (14.3%), fungal hyphae or yeasts were identified by light microscopy; 4 of these were by light microscopy alone. IVCM was performed for 80/112 patients (71.4%); of those, fungal structures were identified in 61 (76.3%). In 28 patients the diagnosis of FK was only made with IVCM (ie, negative on light microscopy, biopsy histology, and culture). A total of 28 cases were tested by panfungal PCR. Six were positive by PCR. Of these, 4 were also positive by both culture and IVCM, 1 was also positive by IVCM, and 1 was also positive by culture. No cases of FK were detected by PCR alone.

### Microbiological Diagnosis

**Figure 3:**
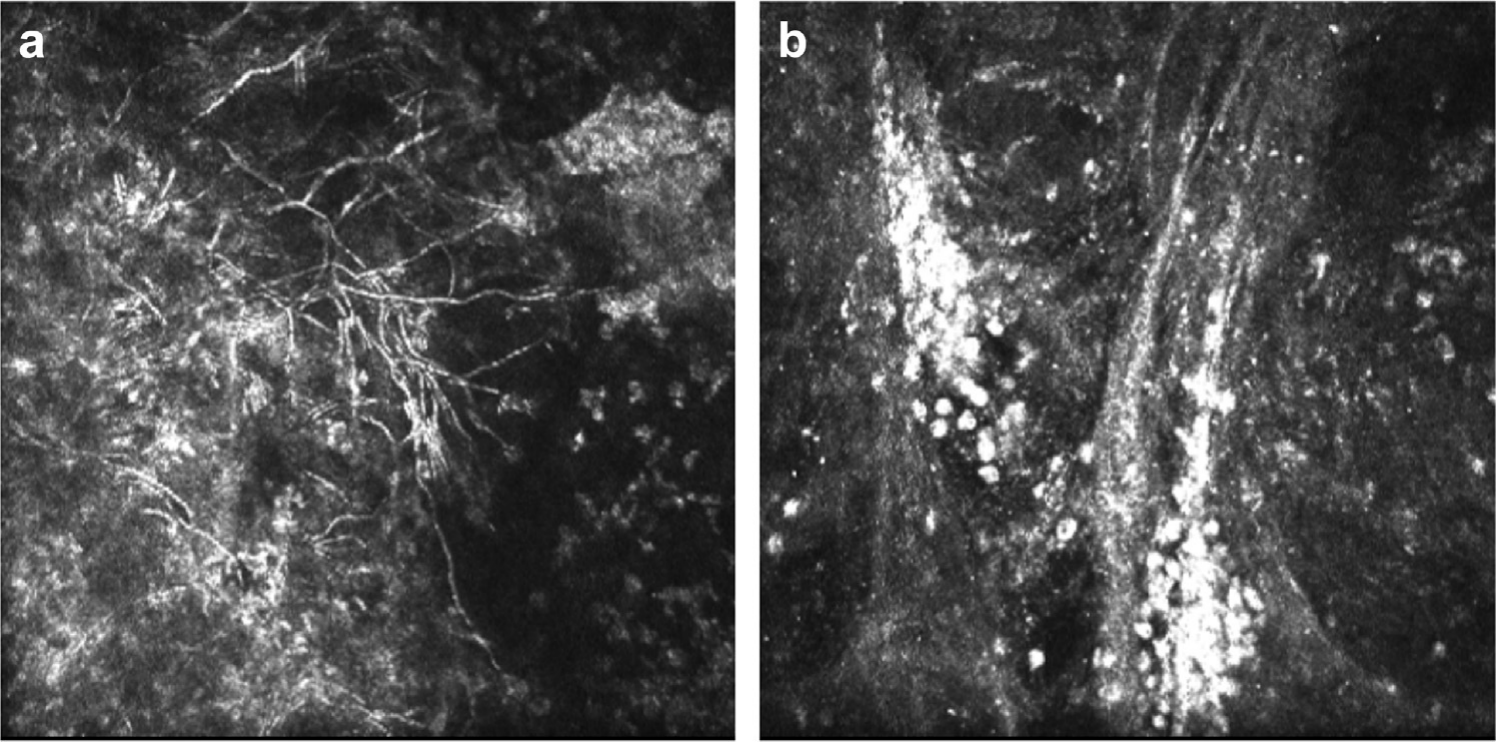
In vivo confocal microscopy images of fungal keratitis: (a) branching filamentary fungal elements, (b) multiple large round yeast bodies.

Overall, 78 of the 112 cases (69.6%) were filamentary fungal infections, 28 of 112 (25.0%) were yeast infections, and 6 of 112 (5.4%) mixed filamentary and yeast infections. There was no evidence of a difference in the pattern of organisms between those acquired in the UK and those acquired outside the UK (*P* = .7). There was evidence (Poisson regression, *P* = .005, Figure 1, Upper right) of an increase in the absolute numbers of cases presenting attributable to filamentary fungi acquired in the UK. However, there was no evidence of such an increase in the number of yeast cases acquired in the UK (Poisson regression, *P* = .3, Figure 1, Upper right), or of an increase in the numbers of filamentary fungi acquired outside the UK (Poisson regression, *P* = .9, Figure 1, Upper right). This suggests that it is the increase in the absolute numbers of filamentary fungi acquired in the UK that is driving the increase in the overall number of FK cases being presented. There was no evidence of a difference in the sex distribution between filamentary fungal and yeast infections (*P* = .6), Table 1. The median age of people with yeast infections was slightly higher than in filamentary cases; however, this was not statistically significant (*P* = .051, Figure 1, Middle left). Of the 61 cases identified by IVCM, 58 had branching filamentary hyphae (of which 27 were culture positive for filamentary fungi) and 3 had large round bodies consistent with a diagnosis of yeast (of which 1 was culture positive for *Candida* spp) (Figure 3).

There were 90 fungal culture isolates (Table 3) from the 79 culture-positive cases. In 11 cases 2 fungal organisms were co-cultured: 7 grew 2 different filamentary organisms and 4 grew a filamentary organism and a yeast. Overall, 30/90 isolates (33.3%) were yeasts and 60/90 (66.7%) were filamentary fungi. *Fusarium* spp were the commonest filamentary fungi isolated (33/79 cases, 41.8%), followed by *Aspergillus* spp (9/79 cases, 11.4%). All the yeast isolates were subspecies of *Candida* (30/79 cases, 38.0%). Bacterial co-infection was present in 20 of the 112 cases (17.9%) (Table 4). In 1 case *Acanthamoeba* was also co-cultured. There was no evidence of a difference in the proportion of filamentary and yeast cases that had a bacterial co-infection (*P* = .5).

### Clinical Course and Treatment

The presenting visual acuity was count fingers or less in 54 (48.7%) of the affected eyes (Table 1). There was evidence that the presenting vision was worse among the cases with yeast infection (*P* = .024). Visions at final follow-up were available in 106 individuals. By the final follow-up there was an improvement in the vision of 74/106 eyes (69.8%), no change in 16/106 (15.1%), and a deterioration in 16/106 (15.1%). The number of eyes with a visual acuity of count fingers or less was 21 (20.0%) at the final follow-up. There was no evidence to suggest that final visual acuities of patients diagnosed by positive cultures compared with patients who had negative cultures (diagnosed by other modalities) were different (*P* = .50). At least 1 corneal graft procedure was performed in 34/112 cases (30.4%): 27 had a therapeutic keratoplasty, 14 had a penetrating keratoplasty, and 5 had a lamellar keratoplasty for restoration of vision. Two patients had a conjunctival flap and 4 eyes were eviscerated.

All patients were treated with topical antifungal agents. During this 8-year period there was a change in the pattern of prescribing for filamentary fungal infections (Figure 1, Middle right). Initially most cases were treated with econazole 1%. From 2009 there was increased use of voriconazole 1%. From 2013 onward there was a move away from voriconazole 1%, to natamycin 5%. Yeast infections were usually treated with topical amphotericin 0.015% or voriconazole 1%. One or more oral antifungal agents were used in 65/112 cases (58%): voriconazole in 48/112 (42.9%), itraconazole in 14/112 (12.5%), and fluconazole in 10/112 (8.9%).

### Risk Factors

Several potential risk factors were reported, including contact lens use, OSD, prior ocular surgery, and a history of trauma (Table 1). Evidence for the associations between the class of fungus isolated (yeast or filamentary) and each of these risk factors for infection are shown in Table 5. The use of contact lenses at the time of onset of symptoms was reported by 64 of 112 individuals (57.1%) (Table 1). There was evidence that filamentary fungal infections were more frequently associated with contact lens use than were yeast infections (Table 5). The types of contact lens used were: soft (for vision), 56/64 (87.5%); therapeutic (bandage), 3/64 (4.7%); and rigid gas-permeable, 4/64 (6.3%). The type of contact lens used was not recorded in one individual. Among the 56 wearing soft (for vision) contact lenses, 11 (19.6%) used daily disposable, 17 (30.4%) used fortnightly disposable, and 25 (44.6%) used monthly disposable. The pattern of wear of contact lenses was not recorded in 3 patients. Although the data on “risk” behavior were not systematically collected, of the soft (for vision) CL users, 4/56 (7.1%) reported overnight use, 9/56 (16.1%) reported swimming in lenses, and 11/56 (19.6%) reported showering in lenses. All 3 cases associated with the use of therapeutic CL had OSD. These lenses were being used for up to 1 month between lens changes.

There was evidence that filamentary fungal infections were less frequently associated with OSD compared to yeast infections (Table 1, Table 5). Of the 25 cases with prior OSD, 6 (24%) had atopic keratitis, 4 (16%) blepharitis, 4 (16%) persistent epithelial defect, 3 (12%) Stevens-Johnson syndrome, 3 (12%) exposure keratitis, 2 (8%) mucus membrane pemphigoid, 1 (4%) dry eye disease, 1 (4%) ectodermal dysplasia, and 1 (4%) neurotrophic keratitis. A persistent epithelial defect was present in 16/112 cases (14.3%) cases prior to the onset of infection, 12 of which had an additional underlying OSD diagnosis.

There was a history of prior ocular surgery in 25 of the 112 cases (22.3%) (Table 1). There was evidence that filamentary fungal infections were less frequently associated with prior ocular surgery than were yeast infections (Table 5). Previous ocular surgery included 8/112 (7.1%) penetrating keratoplasties, 4/112 (3.6%) anterior lamellar keratoplasties, 1/112 (0.9%) limbal stem cell allograft, 3/112 (2.7%) cataract surgeries, 3/112 (2.7%) retinal detachment surgeries, 1/112 (0.9%) phototherapeutic keratectomy, 3/112 (2.7%) laser in situ keratomileusis, 1/112 (0.9%) corneal collagen cross-linking, and 1/112 (0.9%) limbal relaxing incision procedure during cataract surgery.

A history of “trauma” was reported in 13 cases. Three of these were chemical injuries associated with ocular surface complications. Four injures were associated with organic matter and the remainder were associated with nonorganic matter. The proportion of yeast and filamentary fungal infections associated with trauma were similar (Table 1). At presentation, 36/112 (32.1%) cases were on topical corticosteroid (Table 1), 11/112 (9.8%) were on systemic immunosuppression, 5/112 (4.5%) had diabetes mellitus, and 1/112 (0.9%) had a known diagnosis of human immunodeficiency virus (HIV) infection. In the univariate analysis there was evidence that filamentary fungal infections were less frequently associated with steroid use at presentation than yeast infections (Table 5).

In a multivariable logistic regression model comparing risk factors for filamentary fungal infection with yeast infection, filamentary fungal infection was independently associated with contact lens use and OSD was associated with yeast infection (Table 5). The other factors were not statistically significant at the 5% level, and were therefore dropped from the model. Excluding the cases acquired outside the UK, the model produced very similar results in terms of significant factors and their effect size, with no change in inference (Table 5).

### Comparison With the 1994–2006 Series

We reanalyzed a subset of the previously reported study from Moorfields Eye Hospital that included only patients presenting during whole calendar years (January 1, 1994 through December 31, 2006).^27^ This earlier series only included cases that were both UK-acquired and culture positive. It pre-dated the routine use of IVCM. There were 59 culture-positive FK cases during this 13-year period (1994–2006), representing an average of 4.5 cases per year. To compare the 1994–2006 series with the 2007–2014 series, we excluded cases in the later series that were acquired outside the UK or did not have a positive culture. There was evidence of a statistically significant increase in the annual number of culture-positive, UK-acquired FK presenting between 1994 and 2014: Poisson regression *P* = .003 (Figure 1, Lower left). During the 1994–2006 period there were 35/59 (59.3%) yeast infections and 24/59 (40.7%) filamentary fungal infections. *Fusarium* was the cause of 11/59 infections (18.6%). Specific risk factors were associated with the type of microorganism: 18 of 35 (51.4%) yeast infections and 9 of 24 (37.5%) filamentary fungal cases were associated with OSD, whereas 6 of 24 (25.0%) filamentary fungal cases and 1 of 35 (2.9%) yeast cases were associated with CL use.

## Discussion

The diagnosis and management of fungal keratitis is continuing to evolve. In tropical regions fungal keratitis is common and can account for over half of all microbial keratitis cases.^7^, ^8^, ^9^, ^10^, ^11^, ^12^, ^13^, ^14^ In temperate regions, such as the UK, it has been a relatively uncommon diagnosis.^17^, ^27^ Therefore, it may not be considered as a potential diagnosis by clinicians when assessing a new case of suppurative keratitis. However, patterns of infectious diseases change and it is important to monitor the spectrum of isolated pathogens and their associated epidemiology. In this study we report several clinically significant changes in the pattern of fungal keratitis cases presenting to Moorfields Eye Hospital during 8 consecutive years, from 2007 to 2014. This study follows a similar report covering the preceding 13 years (1994–2006).^27^

We found a statistically significant increase in the number of FK cases presenting annually to this hospital during the 2007–2014 study period. Moreover, this change may be part of a long-term increase: the average annualized rate for the 2007–2014 period was 14 cases per year, whereas for the previously reported 1994–2006 period it was 4.5 cases per year.^27^ The numbers of cases presenting during the first 2 years of the present series (2007 and 2008) are comparable to the typical rates in the preceding 13 years. However, there were some differences in the case ascertainment methodology between the 2 series that might explain part of this overall rate difference. The 1994–2006 study only included individuals who had a positive culture and who acquired their infection inside the UK. When we apply the same inclusion criteria to the 2007–2014 series (acquired in the UK with a positive culture) the rate is 9.3 cases per year. This rate is twice that of the 1994–2006 series. It is possible that some of this increase is explained by a modest expansion of the referral base since 2007, with a growth of the UK population, particularly in London and the southeast of England and a slight consolidation of ophthalmic emergency work into fewer centers. However, the increase is of a sufficient magnitude to suggest that there might be an underlying increase in the population level incidence of fungal keratitis, which warrants further investigation with a national survey and case-control study.

In addition to an overall increase in fungal keratitis, there has also been a change in the pattern of organisms identified. Much of the additional fungal keratitis presenting in recent years has been driven by an increase in filamentary fungal infections. We found no evidence for a rise in yeast infections. Historically, fungal keratitis in temperate regions has tended to be dominated by yeast infections.^17^, ^18^, ^19^, ^20^, ^21^, ^22^, ^23^, ^24^, ^25^, ^26^ In the 1994–2006 Moorfields series yeasts accounted for around 60% of all cases.^27^ However, in the 2007–2014 series the situation has reversed, with yeasts identified in 25%, filamentary organisms in 70%, and mixed infections in 5%. The most frequent filamentary organisms identified were *Fusarium* spp, followed by *Aspergillus* spp, which is consistent with many case series from around the world. The increase in cultured filamentary fungi was dominated by an increase in *Fusarium* spp from 18% in the 1994–2006 series to 42% in 2007–2014.

The leading risk factors for fungal keratitis were contact lens use and OSD, although there appears to have been a recent change in their relative contribution. In the 1994–2006 series only 12% of all fungal keratitis cases were contact lens associated.^27^ In the current series this has risen to 57%. The risk factor associations for yeast and filamentary infections were quite distinct. Two-thirds of the filamentary cases were associated with contact lens use, in contrast to only a quarter of the yeast cases. The large majority of these were soft contact lenses. Given the long-term retrospective nature of this study, we were unable to ascertain the specific lenses being used or the related contact lens solutions. However, in view of the experience in 2005 and 2006 when there was an outbreak of fungal keratitis in the United States and Singapore (and a small number of cases in Europe) associated with the use of the ReNu with MoistureLoc (Bausch & Lomb) contact lens solution, it would now seem appropriate that the possibility that this apparent rise in the number of filamentary fungal cases was associated with contact lens use is prospectively investigated.^17^, ^37^, ^38^, ^39^, ^40^, ^41^, ^42^

A prior history of chronic ocular surface disease was much more frequently associated with yeast infections. This observation is consistent with the earlier studies from the UK.^17^, ^27^ A history of anterior segment surgery and prior steroid use were more frequently associated with yeast infections; however, in a multivariable analysis these effects were not independent risk factors for yeast, probably because they were correlated with OSD.

This study illustrates the challenges in making a diagnosis of fungal keratitis and the importance of using several different approaches. The ability to confirm the diagnosis is often held back by relatively low rates of positive cultures. Our ability to diagnose fungal infections has been greatly enhanced by IVCM since 2006. We often find clear IVCM evidence of fungal hyphae in cases that are clinically suspected to be fungal in etiology, but from which an organism cannot be cultured despite repeated attempts, such as in the 28 we report here (Figure 2). However, it is important that the image interpretation be done by an experienced observer to avoid potential confusion with other linear structures, such as corneal nerves or artefacts.^36^ The use of a panfungal PCR assay only entered our routine practice in the last 24 months of the study period. It was therefore not included in the analysis of the diagnostic modalities so that a consistent approach was applied throughout the period. It is noteworthy that during the study period, PCR did not detect any additional cases that were not detected by 1 or more other diagnostic investigations.

We consider the finding of fungal hyphae identified by light microscopy of stained material from the cornea to be diagnostic for fungal keratitis. However, we are concerned that this is an increasingly neglected test. Moreover, special stains such as lactophenol blue or calcofluor white, which may enhance the detection fungal hyphae, may not be routinely performed. Therefore, good communication between ophthalmology and microbiology services is important to maximize the possibility of a positive identification.

There is limited clinical evidence to guide the choice of treatments for FK. During the period being studied (2007–2014), there were 2 shifts in our topical prescribing practice for filamentary fungal infections. First, from 2009 we started using voriconazole 1% instead of econazole 1%. More recently (from 2013) we changed from voriconazole 1% to natamycin 5%. This change was informed by the results of the MUTT1 trial from South India, which found natamycin 5% to be superior to voriconazole 1%, particularly for the treatment of *Fusarium*.^43^ In cases that are unresponsive to either natamycin 5% or voriconazole 1%, our second-line treatment is chlorhexidine 0.2%.^44^ When yeast has been identified we treat with either amphotericin 0.15% or voriconazole 1%. Our experience has been that fungal infections can usually be controlled in the large majority of patients with these agents, indicated by the low number of eyes that needed to be eviscerated in this series. However, very prolonged courses of topical therapy for several months may be needed.^35^, ^45^

Retrospective studies such as this one have several limitations. Firstly, we may not have identified all possible cases and there is a lack of a standardized data recording process. Some patients were referred by other ophthalmic units where they might have received some treatment, potentially making the identification of fungal organisms at our center less likely. We do not have systematic data on pretreatment. Secondly, as it was not a population-based study, we cannot be certain that the changes observed are due to an underlying increase in the incidence of FK, rather than a change in referral patterns or case ascertainment. Thirdly, in the absence of an appropriate control group we are unable to investigate specific risk factors further, beyond reporting the observations of their relative frequency among cases. Despite these limitations, it is possible to draw several clinically significant conclusions of which ophthalmologists practicing in the UK and possibly in other similar countries need to be aware. Firstly, there are reasonable grounds to think that fungal keratitis has become more frequent in recent years. Secondly, there has been a relative increase in the proportion of filamentary fungal cases, which has implications for the choice of antifungal treatment. Thirdly, a greater proportion of cases are now associated with the prior use of contact lenses. Taken together, these observations suggest that more detailed investigation with case-control study methodology and a national surveillance study are warranted.

## Figures and Tables

**Figure:**
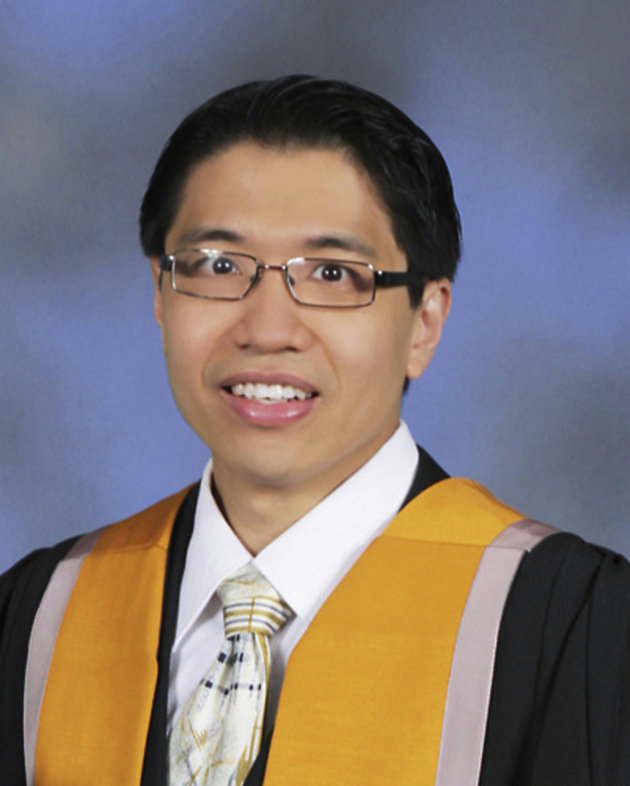
Hon Shing Ong graduated from St Bartholomew's and The Royal London School of Medicine, University of London, United Kingdom. He completed his Ophthalmology specialist training in London. He is currently undertaking a joint research and clinical fellowship in Corneal and External diseases at Moorfields Eye Hospital. He is pursuing a Doctor of Medicine MD (research) degree in ocular surface inflammation and scarring at the Institute of Ophthalmology, University College London.

**Figure:**
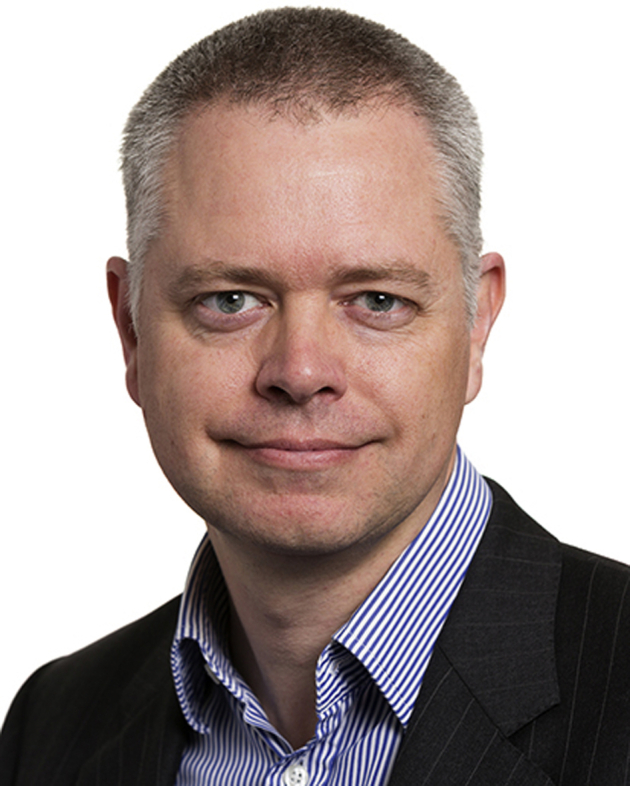
Matthew J. Burton is Reader in International Eye Health / Wellcome Trust Senior Research Fellow at the International Centre for Eye Health, London School of Hygiene & Tropical Medicine, and an Honorary Consultant Ophthalmologist, Moorfields Eye Hospital, UK. His primary academic interest is ocular infections in low- and middle-income countries with a particular focus on trachoma and corneal infections. He studied medicine at Cambridge University and his post-graduate training was in Oxford, and Moorfields Eye Hospital.

**Table 1:** Demographic and Clinical Characteristics of 112 Patients With Fungal Keratitis Presenting to Moorfields Eye Hospital Between 2007 and 2014

Variables	Filamentary	Yeast	Mixed	All
78 (%)	28 (%)	6 (%)	112 (%)
Age, median (IQR)^a^	45.7 (31–56)	49.9 (44–66)	48.6 (28–56)	47.2 (36–57)
Male sex^b^	32 (41.0%)	13 (46.4%)	1 (16.7%)	46 (41.1%)
Risk factors				
Contact lens	52 (66.7%)	7 (25.0%)	5 (83.3%)	64 (57.1%)
OSD	8 (10.3%)	16 (57.1%)	1 (16.7%)	25 (22.3%)
Ocular surgery	11 (14.1%)	14 (50.0%)	0	25 (22.3%)
Trauma	9 (11.5%)	4 (14.3%)	0	13 (11.6%)
Prior steroid use	17 (21.8%)	17 (60.7%)	2 (33.3%)	36 (32.1%)
Presenting VA (n = 111)^c^				
6/5–6/12	14 (18.0%)	2 (7.4%)	2 (33.3%)	18 (16.2%)
6/18–6/60	30 (38.5%)	5 (18.5%)	4 (66.6%)	39 (35.1%)
≤ CF	34 (43.6%)	20 (74.1%)	0	54 (48.7%)
Final best-corrected VA (n = 106)^d^				
6/5–6/12	47 (61.8%)	7 (29.2%)	5 (83.3%)	59 (55.7%)
6/18–6/60	18 (23.7%)	7 (29.2%)	1 (16.7%)	26 (24.5%)
≤ CF	11 (14.5%)	10 (41.7%)	0	21 (19.8%)

CF = count fingers vision; IQR = interquartile range; VA = visual acuity.

a Mann-Whitney test, for the comparison of filamentary and yeast infections: *P* = .051.

b χ^2^ test, for the comparison of filamentary and yeast infections for sex: *P* = .6.

c χ^2^ test, for the comparison of filamentary and yeast infections for presenting VA: *P* = .024; presenting vision was not available in one individual with learning disability.

d χ^2^ test, for the comparison of filamentary and yeast infections for final VA: *P* = .006; final best-corrected VA were only available in 106 individuals (6 individuals were lost to follow-up).

**Table 2:** Regions of the United Kingdom and Other Countries Where Fungal Keratitis Was Acquired, Presenting Between 2007 and 2014

Regions/Countries	n/112 (%)
United Kingdom	91 (81.3%)
Greater London	58 (63.7%)^a^
East of England	17 (18.7%)^a^
Southeast England	9 (9.9%)^a^
Southwest England	3 (3.3%)^a^
West Midlands	3 (3.3%)^a^
Northwest England	1 (1.1%)^a^
Europe	
Malta	1 (0.9%)
Cyprus	1 (0.9%)
Spain	2 (1.8%)
Poland	1 (0.9%)
Americas	
North America (Florida)	1 (0.9%)
Costa Rica	1 (0.9%)
Jamaica	1 (0.9%)
Ecuador	1 (0.9%)
Brazil	1 (0.9%)
Venezuela	1 (0.9%)
Asia	
China	1 (0.9%)
India	2 (1.8%)
Bangladesh	1 (0.9%)
Indonesia	1 (0.9%)
Vietnam	1 (0.9%)
Cambodia	1 (0.9%)
Africa	
Gambia	1 (0.9%)
Nigeria	1 (0.9%)
Algeria	1 (0.9%)

a For UK regions the % values are calculated out of all 91 cases acquired in the UK.

**Table 3:** Analysis of Fungal Species in 79 Cases With a Cultured Isolate, Presenting Between 2007 and 2014

Fungal Species	n/79 (%)
One filamentary fungal isolate	
*Fusarium* sp	26 (32.9%)
*Aspergillus* sp	7 (8.9%)
*Scopulariopsis brevicaulis*	1 (1.3%)
*Acremonium*	2 (2.5%)
*Paecilomyces*	1 (1.3%)
*Rhizomucor*	1 (1.3%)
*Scedosporium apiospermum*	1 (1.3%)
*Cladosporium*	1 (1.3%)
*Curvelaria*	1 (1.3%)
Species not defined	1 (1.3%)
Subtotal	42 (53.2%)
Two filamentary fungal isolates	
*Fusarium* sp + *Acremonieum*	2 (2.5%)
*Fusarium* sp + *Gibberella fujikuroi*	1 (1.3%)
*Fusarium* sp + *Purpureocillium lilacinum*	1 (1.3%)
*Fusarium* sp + *Scedosporium apiospermum*	1 (1.3%)
*Aspergillus* sp + *Chrysosporium*	1 (1.3%)
*Aspergillus* sp + *Scedosporium apiospermum*	1 (1.3%)
Subtotal	7 (8.9%)
Yeasts only	
*Candida* sp	26 (32.9%)
Subtotal	26 (32.9%)
Yeast and filamentary fungal isolates	
*Candida* sp + *Fusarium sp*	2 (2.5%)
*Candida* sp + *Paecilomyces*	1 (1.3%)
*Candida* sp + *Achromobacter xylosoxidans*	1 (1.3%)
Subtotal	4 (5.2%)
Total	79 (100%)

**Table 4:** Bacterial Co-infections Cultured From Cases of Fungal Keratitis, Presenting Between 2007 and 2014

Microorganism	n/112 Percentage (%)
Gram-positive bacteria	
Coagulase-negative *staphylococcus*	7 (6.3%)
*Staphylococcus epidermidis*	2 (1.8%)
*Staphyloccous aureus*	2 (1.8%)
*Staphylococcus capitis*	1 (0.9%)
*Corynebacterium*	1 (0.9%)
*Microbacterium oxydans*	1 (0.9%)
*Brevibacterium*	1 (0.9%)
Gram-negative bacteria	
*Pseudomonas aeruginosa*	3 (2.7%)
*Klebsiella*	1 (0.9%)
*Enterococcus faecalis*	1 (0.9%)

**Table 5:** Analysis of Associations Between Filamentary Fungi and Yeast (Excluding the Mixed Fungal Infection Cases) and Risk Factors for 106 Fungal Keratitis Cases and the 86 Cases Acquired in the United Kingdom, Presenting Between 2007 and 2014

Variable	OR	95% CI	*P* Value
(A) All cases (n = 106)			
Univariate analysis			
Contact lens	6.00	2.26–15.9	.0003
OSD	0.09	0.03–0.24	<.0001
Ocular surgery	0.16	0.06–0.44	.0003
Trauma	0.78	0.22–2.77	.7
Prior steroid use	0.18	0.07–0.46	.0003
Multivariable logistic regression model			
Contact lens	4.35	1.50–12.7	.0070
OSD	0.11	0.04–0.33	.0001
(B) UK-acquired cases only (n = 86)			
Univariate analysis			
Contact lens	6.30	2.16–18.3	.0007
OSD	0.09	0.03–0.28	<.0001
Ocular surgery	0.14	0.05–0.42	.0004
Trauma	1.63	0.32–8.29	.6
Prior steroid use	0.11	0.04–0.32	.0001
Multivariable logistic regression model			
Contact lens	4.42	1.38–14.1	.012
OSD	0.12	0.04–0.40	.0005

CI = confidence interval; OR = odds ratio; OSD = ocular surface disease.
